# Cadmium effects on DNA and protein metabolism in oyster (*Crassostrea gigas*) revealed by proteomic analyses

**DOI:** 10.1038/s41598-017-11894-7

**Published:** 2017-09-15

**Authors:** Jie Meng, Wenxiong Wang, Li Li, Qi Yin, Guofan Zhang

**Affiliations:** 1 Key Laboratory of Experimental Marine Biology, Institute of Oceanology, Chinese Academy of Sciences, Qingdao, 266071 Shandong China; 2 Laboratory for Marine Biology and Biotechnology, Qingdao National Laboratory for Marine Science and Technology, Qingdao, 266071 Shandong China; 3 Laboratory for Marine Fisheries and Aquaculture, Qingdao National Laboratory for Marine Science and Technology, Qingdao, Shandong China; 4 National & Local Joint Engineering Laboratory of Ecological Mariculture, Qingdao, 266071 Shandong China; 5 Marine Environmental Laboratory, HKUST Shenzhen Research Institute, Shenzhen, 518057 China; 6 BGI-Shenzhen, Shenzhen, 518083 China

## Abstract

Marine molluscs, including oysters, can concentrate high levels of cadmium (Cd) in their soft tissues, but the molecular mechanisms of Cd toxicity remain speculative. In this study, Pacific oysters (*Crassostrea gigas*) were exposed to Cd for 9 days and their gills were subjected to proteomic analysis, which were further confirmed with transcriptomic analysis. A total of 4,964 proteins was quantified and 515 differentially expressed proteins were identified in response to Cd exposure. Gene Ontology enrichment analysis revealed that excess Cd affected the DNA and protein metabolism. Specifically, Cd toxicity resulted in the inhibition of DNA glycosylase and gap-filling and ligation enzymes expressions in base excision repair pathway, which may have decreased DNA repair capacity. At the protein level, Cd induced the heat shock protein response, initiation of protein refolding as well as degradation by ubiquitin proteasome pathway, among other effects. Excess Cd also induced antioxidant responses, particularly glutathione metabolism, which play important roles in Cd chelation and anti-oxidation. This study provided the first molecular mechanisms of Cd toxicity on DNA and protein metabolism at protein levels, and identified molecular biomarkers for Cd toxicity in oysters.

## Introduction

Cadmium (Cd) ranks eighth in the priority list of top 20 hazardous substances^1^, and industrialization has greatly increased its input into rivers, estuaries and coastal waters^2^. Marine molluscs, including oysters, can concentrate Cd in their soft tissues at levels of up to 10^4^ times higher than those in seawater, and are thus widely used as biomonitors to detect Cd contamination in coastal environments^3^. Oysters can accumulate metals to phenomenal concentrations, and one critical question is their adaptation and tolerance to metal contaminated environments^4,5^. There is thus a considerable need to understand the molecular mechanisms underlying oysters’ responses to Cd contamination.

Previous studies have mainly investigated single genes in oysters responding to Cd exposure. Metallothioneins (MTs) and several glutathione(GSH)-synthesis-related genes have been identified during Cd chelation^6–8^, and two metal transcription factors (MTF-1/2), which regulate metal chelation, also play important roles under Cd exposure^9,10^. Regarding Cd toxicity, only a few antioxidant enzymes and heat shock proteins (HSP) have been identified^11^. Taylor *et al*.^12^ identified several immune and stress response genes induced by mixed metal exposure using quantitative (real-time) PCR analyses, including HSP70, HSP90, metallothionein and defensin^12^. Recently, transcriptome analysis has been used to identify differentially expressed genes (DEGs) under metal exposure. For example, DEGs involving in the respiratory chain and stress responses have been identified in scallops under copper (Cu) exposure^13^. Previously, we conducted RNA sequence (RNA-seq) analysis under zinc (Zn), Cd, Cu, mercury (Hg) and lead (Pb) exposure in the Pacific oysters *Crassostrea gigas*. Although the results were submitted to the US National Center for Biotechnology Information (NCBI) database, detailed data analysis has not been conducted^14^. These transcriptional level studies only partially contribute to our understanding of stress adaptation, because not all transcripts can be translated, and messenger RNA (mRNA) abundances may not correspond with the protein expression levels due to translation modifications. In molluscs, key proteins expressed under Cd exposure are still unknown, partly due to the lack of a commercial antibody.

Proteomics, with its rapidly expanding analytical tools, provides a method of studying the changes occurring at the proteome levels in response to environmental stressors^15^. Compared to transcription, protein abundances explain over twice as much variation^16^. Proteins represent the molecular phenotype of cells, and have a direct effect on organismal physiology^16^. Two main methodologies are now used in proteomics analysis. In the first method, proteins are separated by two-dimensional electrophoresis, and then identified by either matrix-assisted laser desorption/ionization or electrospray ionisation mass spectrometry after trypsin digestion. In the second method, proteins are first digested and then separated by high-performance liquid chromatography (HPLC), before identification with mass spectroscopy (MS). HPLC-MS has become an important tool in proteomics research, and is widely used in marine molluscs. The isobaric tags for relative and absolute quantitation (iTRAQ) proteomic approach quantifies the proteins based on the HPLC-MS method. In this technique, digested peptides are labelled with amine-specific isobaric tags. Relative protein quantification is achieved by comparing the peak areas of the reporter ions^17^. This method has previously been used to determine the relative protein expression levels in oysters^18^.

Identifying peptides by interpreting MS spectra is a challenging task because there is little information available regarding non-model organisms whose genomes have not been fully sequenced. This results in a relatively small number of proteins being identified, and it is difficult to determine the entire protein expression profile of the organisms. For example, a proteomic study of the clam *Chamaelea gallina* showed that several cytoskeletal proteins changed in response to exposure to Cu and arsenic (As)^19^. Identification was based on homologies with sequences from other organisms, and only the most evolutionarily conserved proteins were identified. Several proteomic studies on metal toxicity have been conducted on oysters. Thompson *et al*.^20–22^ conducted a series of proteomics analysis on Sydney rock oyster (*Saccostrea glomerata*) using 2-dimensional electrophoresis under mixed metals exposure, which revealed that proteomics analysis is a promising approach for assessing the effects of environmental pollution^20–22^. Luo *et al*.^23^ collected *Crassostrea hongkongensis* from three sites with different concentrations of metals, and analysed proteins that presented changes in their relative abundance, referring to these as “differentially expressed proteins” (DEPs), even though the term “differentially abundant proteins” (DAPs) would have been more accurate^23^. However, the majority of the DEPs were not identified because of a lack of oyster genomic information.

Previous studies in oysters have focussed on Cd toxicity and accumulation mechanisms and identified several gene responses to metal exposure. However, several important questions concerning responses to metal exposure continue to persist. First, an important Cd toxicity mechanism is the disturbance of general DNA and protein metabolism via the generation of reactive oxygen species (ROS). While these studies mainly focused on the oxidative stress and identified many genes responsible for antioxidant response, the oxidative stress effects on DNA and protein metabolism have rarely been studied. Secondly, metal accumulation seems to involve a variety of chelation and transportation processes, yet, only one type of metal chelation protein (i.e., metallothionein) has been identified in oysters to data. In the present study, we firstly conducted physiological experiments that revealed Cd was accumulated and induced oxidative stress in oysters. According to enrichment analysis of iTRAQ data, we then found that metal exposure mainly disturbed DNA and protein metabolism in the Pacific oyster *C. gigas*. As a consequence, we set out to study the proteins that are up- or down-regulated in these metabolic processes, as well as the genes that are differentially expressed under Cd exposure, thereby describing two complementary, orthogonal response dimensions. We, furthermore, searched for additional genes and proteins and the underlying metabolic pathways that are related to metal chelation and transport. By conducting these analysis, our objectives were (1) to clarify the effects of oxidative stress on DNA and protein metabolic changes induced by Cd in oysters; and (2) to understand the Cd accumulation mechanism by searching for Cd chelation and transportation genes/proteins. Our results provided a genome-wide characterisation of Cd-responsive proteins in oysters, with a fundamental insight into the complex Cd-responsive regulatory pathways.

## Results and Discussion

### Cd bioaccumulation and induced oxidative stress

**Figure 1:**
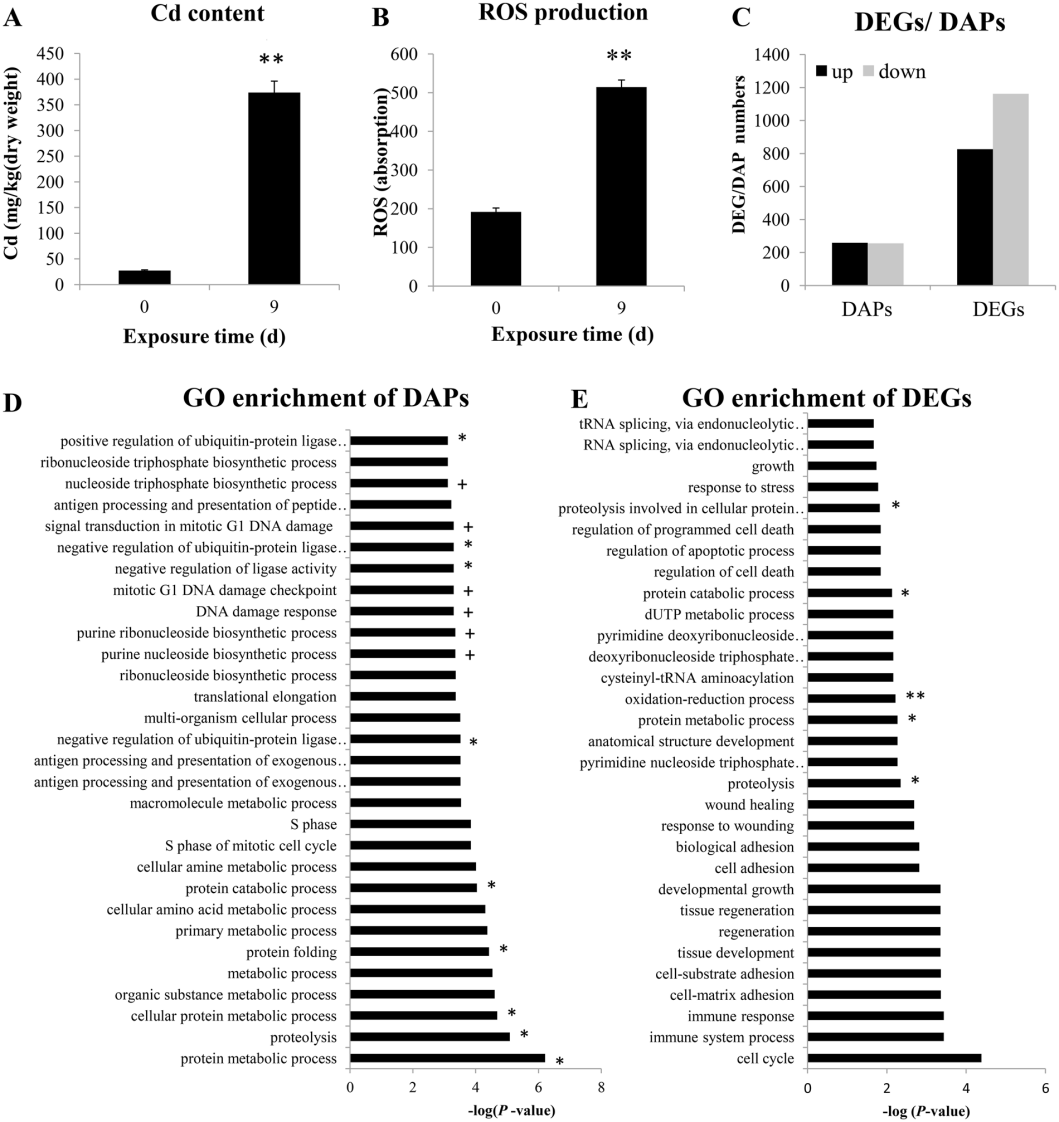
Physiological changes, transcriptome and proteome analysis in oysters under Cd (6 µg/L) treatments. (**A**) Cd concentrations after 9 days of Cd (6 µg/L) exposure. Asterisk (**) indicates significant differences with 0 h exposure (mean ± S.D; N = 6, ANOVA, *P* < 0.01). (**B**) Mean ROS production level (DCF fluorescence in arbitrary unit A.U.) of gills under different Cd exposure times. Asterisk (**) indicates significant differences with PBS control (meanS.D; N = 6, ANOVA, *P* < 0.01). (**C**) Down-regulated proteins/genes (grey) and up-regulated proteins/genes (black) after 9 d of Cd exposure (6 µg/L). DEGs means differentially expressed genes; DAPs means differentially abundant of proteins; (**D**,**E**) GO enrichment analysis of DAPs under 9 d of Cd exposure. The top 30 enriched GO terms in biological process were displaced (multi-level GO terms). The “*” represents the GO terms related with protein metabolism process, “+” represent the GO terms related with DNA damage process.

After 9 days of exposure at 6 µg/L, the Cd concentrations in oyster gills reached values as high as 374 mg/kg (dry weight), and increased by 13.6-fold compared with the control (Fig. 1A). The estimated concentration factor (ratio of tissue concentration to seawater concentration) of Cd in the gills was 6.2 × 10^4^ L/kg, which was comparable to the field measured bioconcentration factor of Cd in oysters^24^. In earlier study, Cd concentrations of 100 mg/g dw were measured in the gills of oysters *Crassostrea hongkongensis* collected from a Chinese estuary moderately contaminated by copper and zinc^24^. The gill concentration of Cd in the oysters measured in this study was about 3.7-fold higher than those measured in the field, and was thus considered to be environmentally relevant.

We conducted physiological experiments to detect reactive oxygen species (ROS) (Fig. 1B), which revealed that ROS production increased significantly after 9 days of Cd exposure as compared to the control. Excess ROS may induce oxidative stress and affect cellular metabolism. Similar results have been obtained in several earlier studies, including on oysters^25^. Cd is unable to generate free radicals directly, and the main mechanisms by which Cd induces oxidative stress is via indirect pathways, such as the induction of nicotinamide adenine dinucleotide phosphate oxidases that bind with thiol groups and replace Fe or Cu ions in Fenton’s reaction at their active sites^26^.

### Characteristics of the proteome and transcriptome databases

Gills were chosen as the target tissue for conducting the proteome and transcriptome analysis described herein thanks to their central physiological role in filter-feeding bivalves^27^. Previous studies have shown that gills constitute a key interface for dissolved metal uptake and are involved in metal storage and detoxification^28^. Analysis of the two biological replicates for the proteome database resulted in 421,868 and 365,464 peptide spectra, which corresponded to 4,672 and 4,964 unique peptides, respectively (Table S1). The peptide number distribution, number of proteins and protein mass distribution are presented in Fig. S1. Only proteins containing at least two peptides and detected in both replicates were used for quantification. Because iTRAQ quantification underestimates the number of “real” fold changes between samples^29^, samples were considered to have a differentially abundant protein (DAP) when there was a significant (*p* ≤ 0.05) and more than 1.2-fold (for up-regulation) or a less than 0.83-fold (for down-regulation) difference between samples, in line with common procedures described in http://www.matrixscience.com/help/quant_statistics_help.html, which have been widely used in many previous studies^30–32^. Consequently, 515 DAPs were identified in oysters exposed to Cd (6 µg/L) for 9 days (Fig. 1C). The reproducibility of the protein quantification was assessed using coefficients of variation (CVs), which were calculated based on the protein ratio obtained from the two replicates. When the threshold of the CV value was set to 30%, more than 80% of the quantified proteins were below this value (Fig. S2), indicating that the quantification was highly reproducible^17^.

The characteristics of the transcriptome libraries (control and 9 days) are summarised in Table S2. The transcripts detected 99% of the proteins (Fig. S3). A total of 1988 DEGs were detected after 9 days of exposure compared with the control. The DEGs and DAPs were subjected to a Clusters of Orthologous Groups (COG) analysis, which displayed the different patterns (Fig. S4). According to the analysis of the DAPs accounting for the percentage (%) of total protein numbers for the different categories, the top five largest percentage COG groups were A (RNA processing and modification), U (Intracellular trafficking, secretion, and vesicular transport), Z (Cytoskeleton), O (Post-translational modification, protein turnover, chaperones), B (Posttranslational modification, protein turnover, chaperones). However, at the mRNA expression level, the top five largest percentages of COG groups were B, S (Function unknown), Z, Q (Secondary metabolites biosynthesis, transport and catabolism), M (Cell wall/membrane/envelope biogenesis).

### Gene Ontology (GO) enrichment analysis revealed the Cd response mechanism

For the DAPs and DEGs, we conducted a GO analysis to classify gene and protein functions in terms of three GO ontology processes, including biological processes (BP), molecular functions (MF) and cellular components (CC). GO is loosely hierarchical, with ‘child’ terms being more specialized than their ‘parent’ terms. The terms directly related with three GO ontology process (BP, MF and CC) were defined as GO level 2. The GO classification at level 2 displayed the same distribution for both the RNA-seq and iTRAQ data (Fig. S5). The most enriched biological process GO terms were ‘metabolic process’, ‘cellular process’ and ‘single organism process’. The most enriched cellular component process GO terms were ‘cell part’, ‘cell’ and ‘organelle’. The most enriched molecular function process GO terms were ‘catalytic activity’ and ‘binding activity’.

We investigated the top 30 enriched GO terms with the lowest *P*-values in biological processes for the proteomic data. A total of 9 GO terms relating to protein metabolism were enriched after 9 days of Cd exposure (Fig. 1D, Table S3), including ‘protein folding’, ‘protein targeting to ER’, ‘proteolysis process’ and ‘protein ubiquitination process’. In addition, six terms related to DNA metabolism were enriched (Fig. 1D, Table S3), including ‘DNA damage’ and ‘DNA damage response’. These analyses strongly suggested that exposure to Cd concentrations of 6 µg/L in our experiment may interfere with protein and DNA metabolism in the oysters. The same phenomenon was also reported in mammals^33^. Excess Cd can indirectly produce reactive radicals by suppressing free radical scavengers and detoxifying enzymes, which lead to oxidative stress. At this state, the increased formation of ROS overwhelmed the body’s antioxidant protection, causing DNA damage, protein modifications and other effects^34^. The increased ROS production under Cd exposure indicated that Cd induced oxidative stress in the oysters.

We further investigated the top 30 (with the lowest *P*-values) enriched GO terms for the transcriptome data (Fig. 1E, Table S4). The protein metabolism process, including ‘proteolysis’ and ‘protein catabolic process’, was enriched, according to the iTRAQ data. In addition, ‘oxidation-reduction process’ was enriched, which further indicated that excess Cd induced the oxidative stress response in oysters. One of the most important mechanisms of Cd-induced redox responses was by binding to sulphydryl groups of proteins and depleting GSH^34^. GSH metabolism was also affected by Cd exposure.

Overall, GO enrichment analysis revealed that exposure to Cd concentrations of 6 µg/L in our study may disturb protein folding, proteolysis and DNA damage in oysters. However, we only displayed top 30 enriched terms with the lowest *P*-value and focused on DNA and protein metabolism process based on the objective of our study. There are potentially other physiologically important responses, which are not discussed in our study.

### Effects of Cd exposure on DNA metabolism

Cd exposure negatively affected DNA metabolism by causing DNA damage and inhibiting DNA repair. In molluscs, several studies have reported cellular DNA damage under metal exposure^35^; however, the genes involved have not been identified.

#### DNA repair process

**Figure 2:**
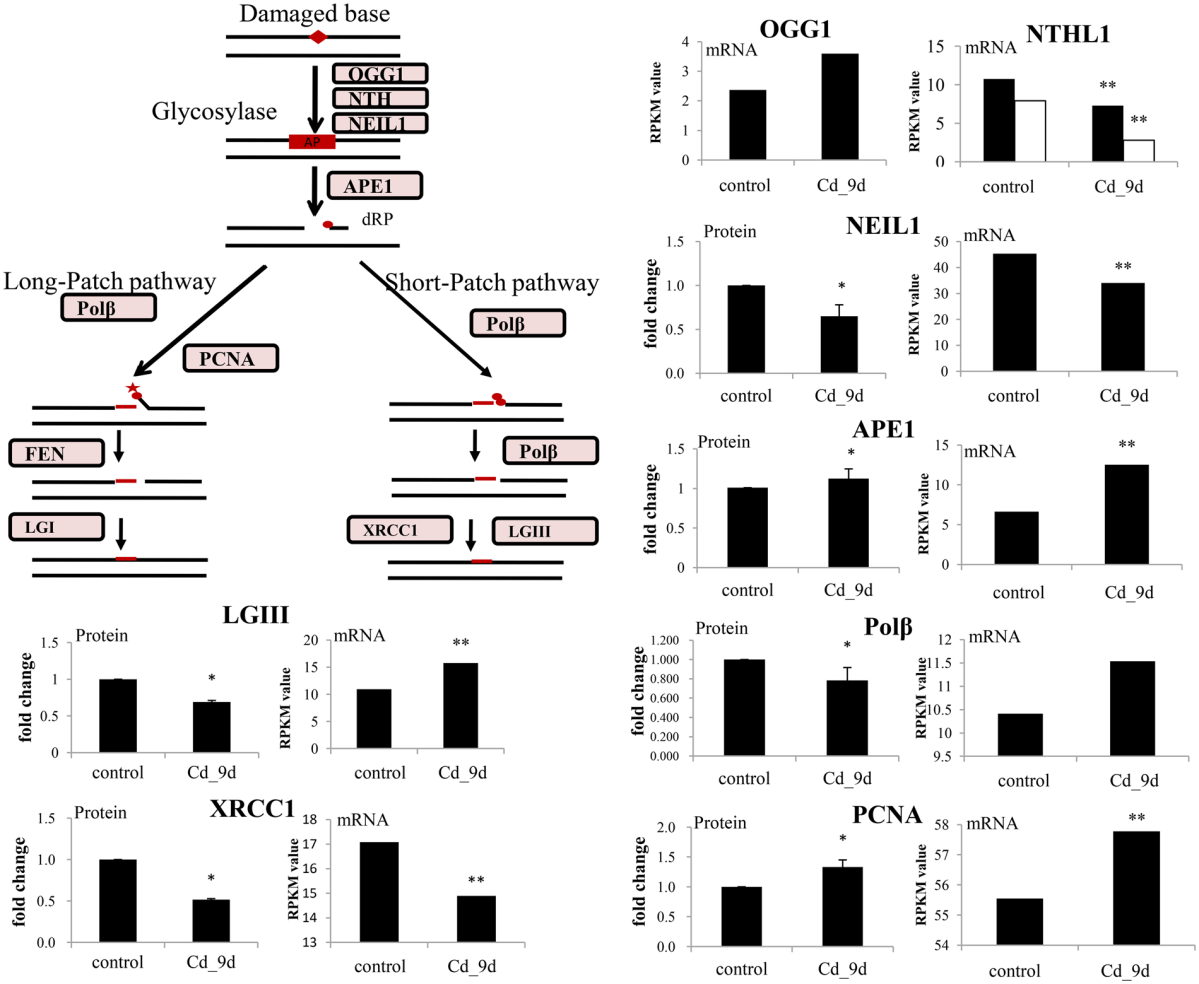
Overview of Cd toxicity in base excision repair (BER) metabolic pathway in oysters. The mRNA expression was measured by RPKM (reads per kilobase per million mapped reads) from RNA-seq data. The significance was calculated with Chen’s methods^87^, with FDR < 0.01 (**). For protein abundance, the fold changes compared with control group (0 h after exposure) were displayed after 9 days of Cd treatment (*indicates *P* ≤ 0.05 and fold changes ≥1.2). The error bars represent the standard deviation (n = 2) of protein abundance detected in two replicates of iTRAQ results. OGG1: 8-oxoguanine-DNA glycosylase; NTH1: endonuclease III; NEIL1: endonuclease VIII-like 1; APE: AP-endonuclease; PNK: polynucleotide kinase; DNA Pol β: DNA polymerase β; PCNA: Proliferating cell nuclear antigen; FEN: Flap endonuclease; Lig I: DNA ligase I; XRCC1: X-ray repair cross-complementing 1; DNA ligase III: LGIII. For NTHL1, the black and white bar represent two copies of this gene/protein.

In mammals, Cd affects the DNA repair system by inhibiting the expression of key enzymes. In molluscs, little is known about DNA repair systems, although they do exist^36^. One important DNA repair system dealing with Cd toxicity is the base excision repair (BER) system. We found six key proteins in the BER pathway which exhibited significantly different expression levels under Cd exposure, indicating that Cd affected the BER system in the oysters. We systemically analysed the key protein abundance changes in four BER metabolic phases: (1) base excision by a DNA glycosylase; (2) apyrimidinic/apurinic (AP)-endonuclease (APE) or polynucleotide kinase (PNK) to generate a 3′ OH terminus at the damage site; (3) repair with a DNA polymerase; (4) DNA nick-sealing using a DNA ligase. In the first two steps, five proteins in two sub-pathways were identified in the oyster genome, including endonuclease VIII-like 1(NEIL1)-PNK and 8-oxoguanine-DNA glycosylase (OGG1)/endonuclease III (NTH1)-APE1. The abundance of NEIL1, which is the initial glycosylase that catalyses βδ elimination at AP sites in the NEIL1-PNK metabolism pathway, was 0.65-fold of the control (*p* < 0.05) after Cd exposure (Fig. 2). Its mRNA expression was 0.75-fold of the control (*p* < 0.05) (Fig. 2). These observations were also reported in mammals^37^. Excess Cd could bind to NEIL1 and alter its secondary structure, strongly inhibiting the repair of a common cytosine oxidation product, mutagenic 5-hydroxyuracil^38^.

Key proteins in the OGG1/NTH1-APE metabolism sub-pathway were also expressed under Cd exposure. OGG1 is the initial glycosylase that catalyses β elimination at AP sites and produces 3’ phospho α, β-unsaturated aldehyde^38^. In the oyster genome, we only identified one copy of the OGG1 gene; its mRNA expression increased slightly (1.19-fold, *p* > 0.05) but not significantly under Cd exposure (Fig. 2). In mammals, OGG1 mRNA expression exhibits time- and dose-dependent down-regulation under Cd exposure, and is a useful biomarker for assessing oxidative stress^39^. The unchanged expression levels indicated that there may exist different regulatory mechanisms in molluscs depending on different exposure times and concentrations. NTH1 is another important glycosylase, and its mRNA expression was 0.35 fold compared with control (*p* < 0.05) after Cd exposure (Table S4). This indicates that Cd exposure inhibits NTH1-mediated DNA glycosylase/AP-lyase, rather than OGG1-mediated DNA glycosylase/AP-lyase. However, the protein abundance of OGG1 and NTH1 were not found in the iTRAQ database. APE1 is an endonuclease and a 3′ exonuclease, and is important in the repair of alkylated bases generated by OGG1 and NTH1. Both mRNA expression (1.9-fold) and protein abundance (1.21-fold) of APE1 increased significantly (*p < *0.05) under Cd exposure (Fig. 2). In previous studies, both decreased and increased APE1 protein expression has been observed under Cd exposure in mammalian cells, depending on the levels of manganese (Mn), nickel and magnesium (Mg)^40^. For example, a reduction in APE expression in human cells (HCT165) was reported under excess Cd; however, this can be completely restored by excess Mg^40^.

We also investigated the last two steps of BER: gap-filling and ligation. In mammals, it has been reported that Cd inhibited related protein abundance, including DNA polymerase β (polβ), poly(ADP-ribose) polymerase (PARP) and DNA ligase I (Lig I). However, the inhibitory effects are different in mammalian cell lines^40^. Further studies are required to characterize the precise components of DNA repair affected by Cd. DNA polβ, X-ray repair cross-complementing 1 (XRCC1) and DNA ligase III (LGIII) are the core enzymes in the short-patch BER pathway^41^. All three proteins’ abundances significantly decreased under Cd exposure. DNA polβ fills gaps with the correct nucleotide, and XRCC1 is important in the short-patch BER pathway because it interacts with LIGIII, which is a core BER enzyme. A lack of these three enzymes would result in abasic sites, single-strand breaks with blocked termini, single-nucleotide gaps and ligatable DNA nicks^42^. The decreased abundance of these three proteins indicated that Cd inhibited gap-filling and ligation in the short-patch BER pathway in oysters. The mRNA expressions of XRCC1 also significantly decreased (Fig. 2). However, LGIII mRNA expression significantly increased under Cd exposure, which was not the case for its protein expression, indicating that post-transcriptional modifications may have occurred for this gene. In the long-patch BER pathway, proliferating cell nuclear antigen (PCNA) acts with DNA polβ to synthesise relatively long DNA chains^41^. The decreased DNA polβ expression indicated that the gap-filling process was also inhibited in the long-patch BER pathway in oysters. However, the protein abundance of PCNA increased significantly (1.33-fold, *P < *0.05) under Cd exposure. PCNA enables the functioning of both replicative and translation synthesis DNA polymerases, and stimulates other enzymes involved in DNA replication and repair^43^. Increased PCNA abundance may represent an increase in DNA replication rather than an increase in DNA repair.

Overall, we found that Cd exposure inhibited NEIL1 and NTH1 glycosylase, and gap-filling and ligation in the short-patch BER pathway were also inhibited. These candidate genes/proteins could be used as biomarkers to detect excess Cd in oysters. However, several key proteins, including OGG1, APE1 and PCNA, were not inhibited, which were not identified in previous studies on mammals, indicating different regulation mechanisms in oysters.

#### DNA damage response

In molluscs, several comet assay experiments showed that Cd caused DNA damage. For example, Cd caused DNA damage in *Mytilus edulis* by breaking DNA strands and by oxidation^44^. However, the molecular biomarkers have not been studied. We found that three important DNA replication-related proteins, Cdt1, minichromosome maintenance protein (MCM4) and PCNA, were enriched in the ‘DNA damage response’ term (GO:0000077) (Fig. S6). Their high protein abundance under Cd exposure may induce the DNA re-replication process^43^. The same mechanism was also observed in *M. edulis* under benzo[α] pyrene exposure^45^. Although these genes’ functional mechanisms require further investigation in molluscs, their protein abundance changes may be used as biomarkers to detect the excess Cd.

### Effects of Cd exposure on protein metabolism

The results of the GO enrichment analysis suggested that excess Cd affected protein folding (the refolding of abnormal proteins) and protein ubiquitination (the removal of unrepaired proteins) in oysters. Previous studies on molluscs have investigated several protein-folding-related genes under Cd exposure, such as HSP70 and HSP90^33^, but ubiquitination-related proteins have not been studied.

**Figure 3:**
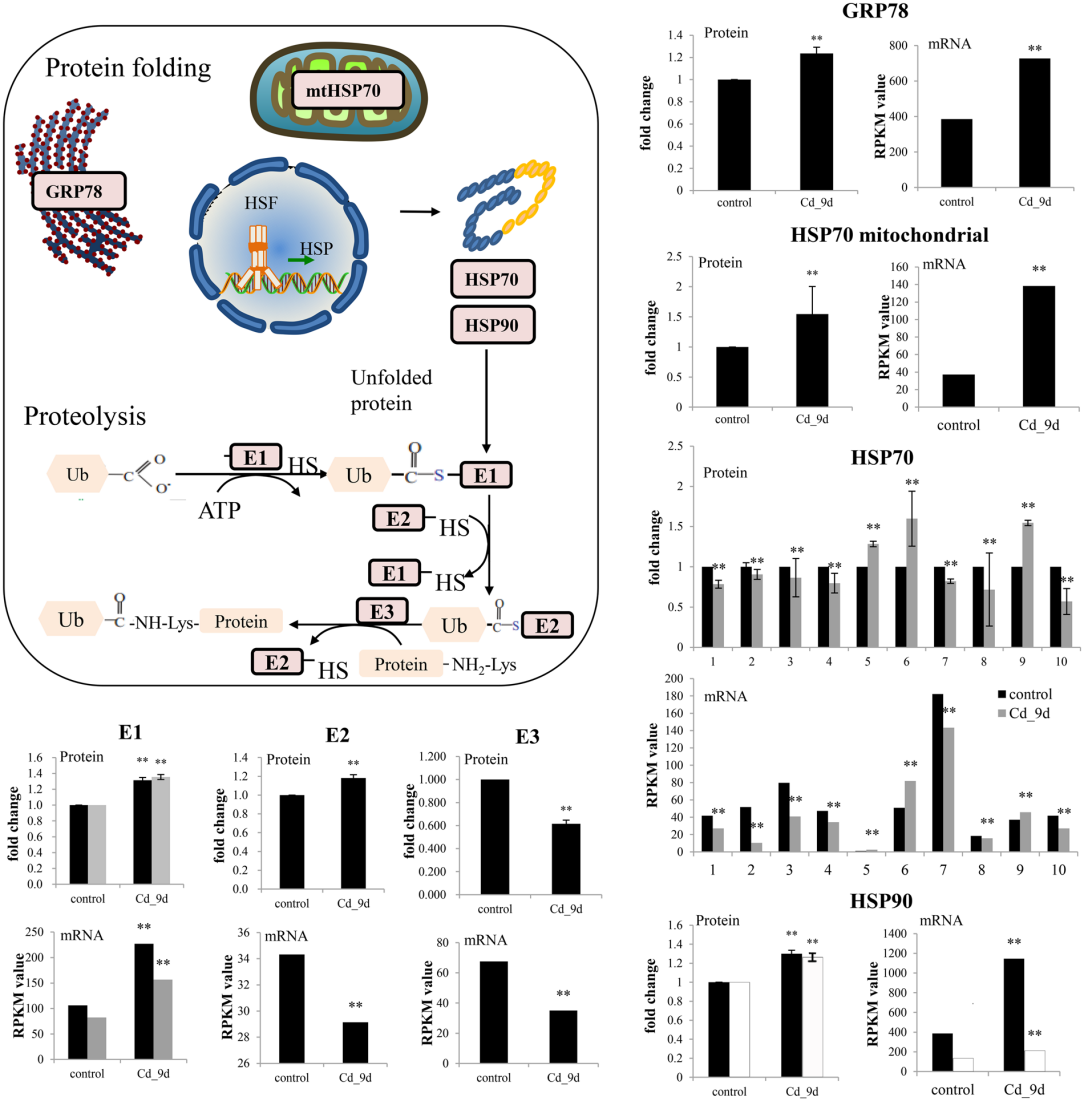
Overview of Cd toxicity in protein folding and proteolysis process in oysters. The mRNA epxressions were measured by RPKM from RNA-seq data. The significance was calculated with Chen’s methods, with FDR < 0.01 (**). For protein abundance, the fold changes compared with control group (0 h after exposure) were displayed after 9 days of Cd treatment (*indicates *P* ≤ 0.05 and fold changes ≥1.2). The error bars represent the standard deviation (n = 2) of protein abundance detected in two replicates of iTRAQ results. mtHSP70: mitochondrial heat shock protein 70; HSP90: heat shock protein 90; GRP78: glucose-regulated protein 78; HSF: heat-shock factors; E1: E1 ubiquitin-conjugating enzymes; E2: E2 ubiquitin-conjugating enzymes; E3: E3 ubiquitin-conjugating enzymes. For HSP90, the black and white bar represent two copies of this gene/protein.

Analysis of the DAPs suggested that several HSP gene family related proteins displayed different abundance, including 11 copies of HSP70, 9 copies of HSP60, 2 copies of HSP90, 5 copies of DnaJ and 2 copies of 78-kDa glucose-regulated proteins (GRP78) (Fig. 3). These proteins are important components of the cellular network of molecular chaperones and folding catalysts, and participate in a wide variety of protein-folding processes in the cells by transient associations of their substrate-binding domain. HSP70 and HSP90 are in protein families that include universal cytosolic chaperones, while HSP60 and GRP78 are predominantly found in mitochondria and the endoplasmic reticulum (ER). Their high mRNA expression and protein abundance indicated that excess Cd induced protein folding in the cytosol, mitochondria and ER (Fig. 3). One copy of GRP78, which was a quintessential ER stress indicator, significantly increased its mRNA expression (1.9-fold, *p < *0.05) and protein abundance (1.23-fold, *p* < 0.05) under Cd exposure (Fig. 3), indicating that Cd was also a strong ER-stress inducer. The high gene numbers and diverse expression patterns of HSP70 may help oysters to maintain cellular homeostasis under Cd exposure.

We also investigated the key genes and proteins in ‘protein ubiquitination process’ (GO: 0016567). Protein ubiquitination is a highly conserved pathway, and plays an important role in the selective degradation of specific cellular proteins^46^. E1, E2 and E3 enzymes help the substrate protein being tagged by multiple ubiquitin molecules, and then degraded by a 26 S proteasome. Protein ubiquitination modulates metal induced toxicity in model species^47^. For example, in yeast, mutants deficient in specific ubiquitin-conjugating enzymes are hyper-sensitive to Cd^48^. However, no study has been conducted to analyze the Cd toxicity to the protein ubiquitination process in molluscs. We found that ubiquitin-conjugating proteins, including E1, E2 and E3, were enriched in ‘protein ubiquitination process’ (GO:0016567). The protein abundance of E1 and E2 significantly increased after Cd exposure (Fig. 3), indicating that excess Cd triggered the protein ubiquitination response. However, for E2, there was an opposite response for mRNA expression and protein abundance, with mRNA decreasing upon Cd exposure whereas protein abundance increased. This might be due to mRNA changes occurring faster than protein level protein changes. Both the mRNA expression and protein abundance of E3 significantly decreased after Cd exposure. Several possibilities may explain such decrease. Some E3 enzymes contain sulfhydryl groups in the active sites and may be oxidized in the presence of high Cd concentrations^49^. The depletion of ATP by severe oxidative stress is also known to induce the decline in levels of ubiquitin conjugates^49^. Finally, sustained oxidative stress may impair proteasome activity and reduce the degradation of ubiquitinated substrates, contributing to the decreased expressions of ubiquitin conjugate enzymes^50^.

### Effect of Cd on oxidative stress responses

Metal stress can accelerate the generation of cellular ROS, and organisms have evolved robust antioxidant defence mechanisms. GO enrichment analysis suggested that ‘oxidative stress response’ was enriched under Cd exposure. The oxidative stress response mainly involves key antioxidant enzymes, including catalase (CAT), superoxide dismutase (SOD) and some key enzymes in the GSH metabolism process.

#### GSH metabolism

The high affinity of Cd for thiol groups causes an imbalance in the available glutathione (GSH) pool, which affects the GSH-oxidised GSH (GSSG) cycle. This is the most important mechanism of oxidative stress induced by Cd, thus GSH is considered one of the most important antioxidant agents. Moreover, GSH is also the cofactor of many enzymes that catalyse metal detoxification and excretion. Thus, the GSH production and consumption were of particular interest in our study.

**Figure 4:**
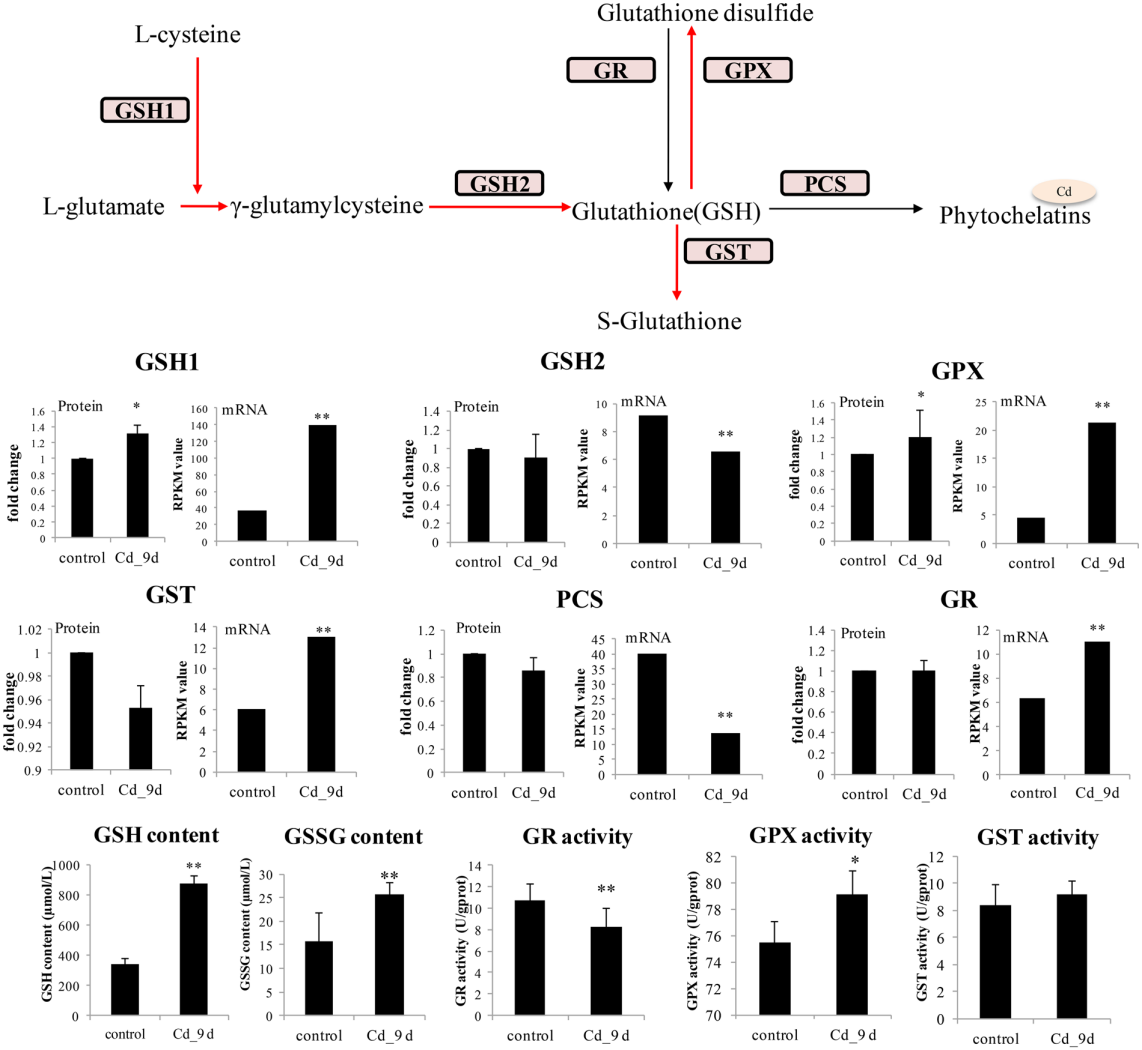
Expressions and activities of key enzymes in the glutathione (GSH) metabolic pathway. Diagram of the GSH metabolic pathway, showing the key enzymes regulating GSH synthesis and catabolism. The mRNA epxressions were measured by RPKM from RNA-seq data. The significance was calculated with Chen’s methods, with FDR < 0.01 (**). For protein expressions, the fold changes compared with control group (0 h after exposure) were displayed after 9 days of Cd treatment (*indicates *P* ≤ 0.05 and fold changes ≥1.2, n = 2). The error bars represent the standard deviation (n = 2) of protein abundance detected in two replicates of iTRAQ results. The GSH and GSSG contents, GPX, GR and GST activities were also measured and the significance was based on t-test (**indicates *P* ≤ 0.01, *indicates *P* ≤ 0.05, n = 6). GSH1: gamma-glutamyl cysteine synthetase; GSH2: glutathione synthetase; GR: glutathione reductase; GPX: glutathione peroxidase; PCS: phytochelation synthase; GST: glutathione S-transferase.

First, we analyzed the GSH synthesis related enzymes, including gamma-glutamyl cysteine synthetase (GSH1) and glutathione synthetase (GSH2). GSH1 was the GSH synthesis rate-limiting enzyme and increased significantly at the mRNA (3.8-fold) and protein (1.3-fold) level after Cd exposure (Fig. 4). Its high expression also induced GSH accumulation (2.6-fold increase) and provided a sufficient amount of GSH under Cd exposure. Similar results were obtained in rat proximal tubule cells^51^. It was proposed that the increasing GSH contents provided a protective response to Cd toxicity. However, the mRNA expression of GSH2 decreased, while its protein abundance did not change significantly under Cd exposure. It has been proposed that GSH1 and GSH2 display different sensitivity to Cd exposure and are induced at different Cd concentrations^52^. Also, the decreased expressions of GSH2 and increased GSH accumulation may indicate that GSH2 was not the limiting enzyme for GSH synthesis in our results.

GSH is very important in redox sensing through the cellular GSH/GSSG ratio, which is controlled by glutathione peroxidase (GPX) and glutathione reductase (GR). GPX could eliminate ROS ions (hydrogen peroxide) and catalyse GSH to form GSSG. In our study, GPX significantly increased at the protein (1.2-fold, *p < *0.05) and mRNA (4.7-fold, *p < *0.05) levels under Cd exposure. Also, its enzyme activities increased by 1.1-fold (Fig. 4). However, GR, catalyzed the reduction of GSSG to GSH by using a reduced form of NAD(P), did not change significantly at protein level. Also, its enzyme activity was 0.76-fold diminished compared with control (0 h) under Cd exposure (Fig. 4). This corroborates previous studies which have indicated that Cd has a negative effect on the GR expression after prolonged exposure. The different expression patterns of GPX and GR may induce the increased contents of GSSG^53^, which was also observed in our results (increased 1.7-fold).

Besides its role as an antioxidant molecule, GSH also plays an important role in metal chelation. Glutathione-S-transferase (GST) catalyses GSH to detoxify metals by conjugation and forms metal-GSH compounds in mammals, which has been widely studied. However, in aquatic organisms, the GST-MRP (multi-drug resistant associate protein) pathway appears to play an important role in multi-xenobiotic resistance. In several fish species, GST-MRP was shown to play important roles in Cd chelation. In our results, both the protein abundance and enzyme activity of GST did not change significantly (Fig. 4), which suggests that this was not the major metal chelation pathway in oysters. GSH is also catabolised by phytochelatin synthase (PCS), and phytochelatin (PC) is an important metal-binding protein in plants and many invertebrates^54^. However, PCS protein abundance did not change significantly under Cd exposure (Fig. 4), while its mRNA expression decreased significantly (Fig. 4). In previous studies, phytochelatins have been shown to have a high affinity for binding with metals in most plants and invertebrates, particularly for Cd^55,56^. Also, PC is the most important protein for Zn chelation in oysters (Meng J, unpublished data). However, the decreased expression observed in our study suggests that there were different regulation mechanisms for PCS under Cd exposure in oysters. Also, unlike in plants, PC may have different ion specificities in oysters.

In order to investigate the Cd chelation mechanism in oysters, we analyzed another important group of Cd chelation proteins: metallothioneins (MTs), which are the only Cd chelation proteins currently identified in oysters^57,58^. According to a genome-wide search, five MTs genes were found, which increased significantly in their mRNA expression upon Cd exposure (28.3, 29.5, 25.2, 1.8 and 2.9-fold respectively, Fig. S7), which indicated their important roles in the chelation process. However, measurement of MTs protein abundance in molluscans have been hampered by the lack of a suitable antibody and in our study, MTs were not quantified in iTRAQ analysis because of technological limitations in identifying the corresponding chromatograms. Using western blot analysis, we observed significant changes in protein abundance of MT2, which also displayed the largest changes at mRNA expression level under Cd exposure (Fig. S7), corroborating the important roles of MTs in Cd chelation in oysters.

Overall, the changed protein abundances of GPX and GR under Cd exposure indicated the important roles of GSH in Cd antioxidation system. However, the unchanged PCS and GST protein abundance indicated its minor roles in metal chelation, whereas the high expressions of MTs revealed its important roles in the Cd chelation process in oysters.

#### Other antioxidant enzymes

Other antioxidant enzymes, including SOD and CAT, also play important roles under metal exposure. For example, in the gastropod *Achatina fulica*, both SOD and CAT activity are inhibited under Cd exposure^59^. However, in freshwater clams (*Corbicula fluminea*), excess Cd increased the SOD expression and decreased the CAT expression^60^. These studies mainly focused on enzyme activity changes and mRNA expression. In the present study, we investigated antioxidant enzyme expression at both the mRNA expression and protein abundance levels under Cd exposure. After Cd exposure, the protein abundances of Cu/Zn-SOD, Mn-SOD and CAT significantly decreased (Table S5); similar changes to the mRNA expression level were observed for Cu/Zn-SOD and Mn-SOD. These decreases have been reported in a previous study in oysters after 3 days of Cd exposure^61^. The over-accumulation of Cd beyond a certain level may cause oxidative stress that dramatically lowers the metabolic capacity, resulting in a decrease in antioxidant enzyme activity. However, CAT mRNA expression levels significantly increased after 9 days of Cd exposure, indicating the presence of a post-transcriptional modification mechanism.

### DAPs and DEGs analysis identified new Cd transporters

Cd is a non-essential metal and does not have specific transporters^50^. In mammalian studies, it is generally considered that Cd uptake occurs via pathways that are also used by essential trace metals. Several proteins, including MRPs, ZIPs, calcium channels, Nramp2 and the DMT1 transporter, have been identified as the main Cd transporters in mammals^62^. However, the precise transportation mechanism across cell membranes is almost unknown in molluscs^63^. We found that only three transporters (ZnT1, ATPase7A and MRP) exhibited differential expression levels under Cd exposure.

**Figure 5:**
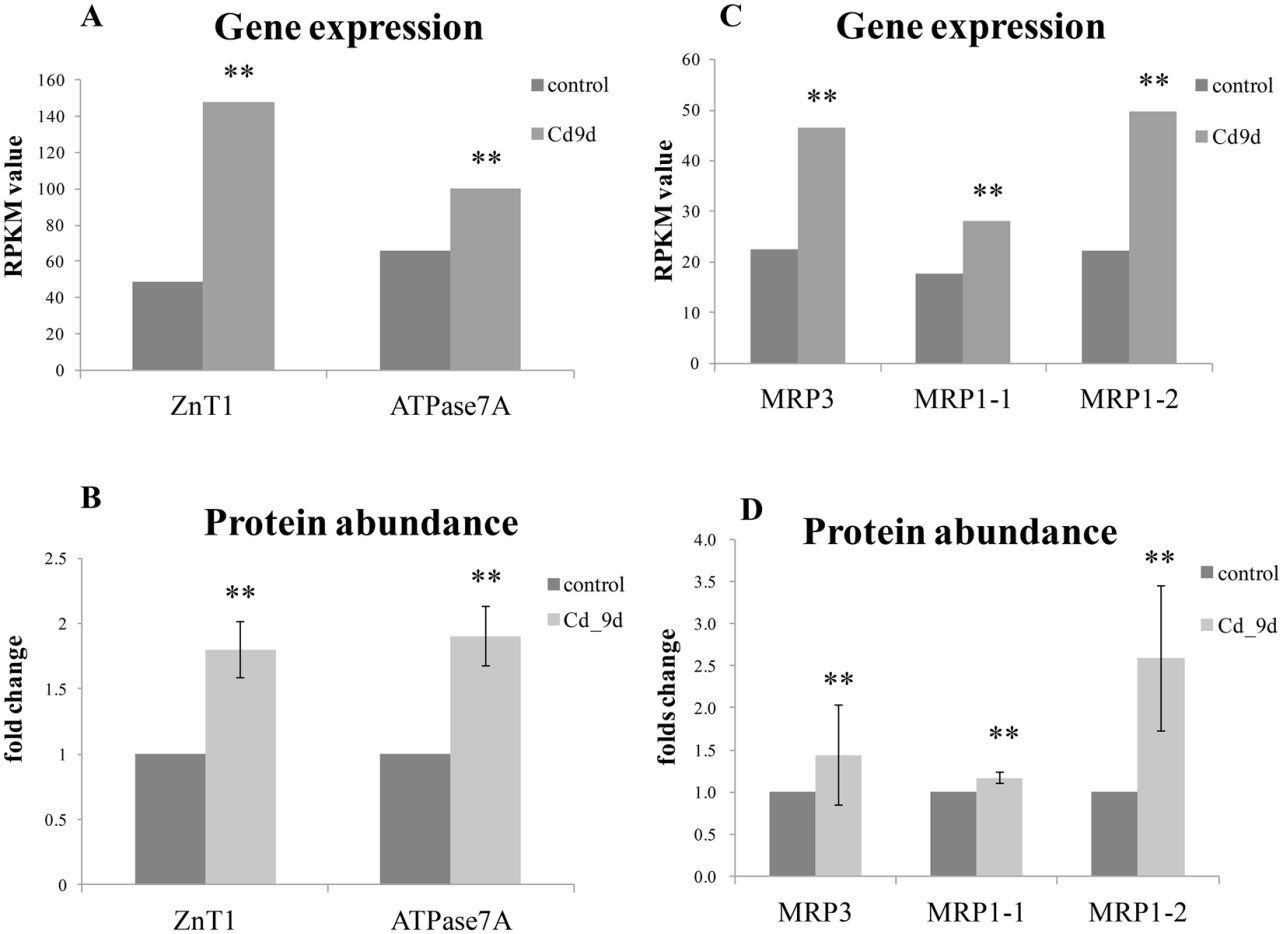
The mRNA expression and protein fold changes of the key genes/proteins participating in Cd transport process after 9 d of Cd exposure in oysters. The mRNA expression was measured by RPKM from RNA-seq data. The significance was calculated with Chen’s methods, with FDR < 0.01 (**). For protein abundance, the fold changes compared with control group (0 h after exposure) were displayed after 9 days of Cd treatment (*indicates *P* ≤ 0.05 and fold changes ≥1.2, n = 2). ZnT1: zinc transporter 1; ATPase7A: MRP: multi-drug resistance associated protein.

The protein abundance and mRNA expression levels of ZnT1 increased 2.0- and 3.0-fold, respectively, after Cd exposure, indicating the important role of ZnT1 in Cd uptake (Fig. 5). Similar expression changes have been reported in plants^64^ and mammals^50^. The results of our previous study suggested that oyster ZnT1 located in cellular membrane and its expression was regulated by metal transcription factor 1 (MTF-1), which is an intracellular Zn sensor^10^. In mammals, excess Cd could also lead to MTF-1 activation to induce the concomitant release of Zn^65^. This may be the main mechanism involved in the induction of ZnT1 expression under Cd exposure.

ATPase7A, which belongs to a metal-transporting gene family, plays an important role in Cu homeostasis in plants and mammals^66^. In our study, there was a 1.9-fold increase in ATPase7A protein abundance and transcriptional level expressions under Cd treatment (Fig. 5). However, a mammalian study found that Cd is not exported via ATPase7A^66^. Because excess Cd could displace Zn and Cu from metallothionein, we propose that Cu homeostasis was disrupted under Cd exposure, and in turn induces high ATPase7A expression.

Three MRP copies significantly increased at transcriptional levels and protein abundance under Cd exposure, consistent with previous studies^67^ (Fig. 5). MRPs are involved in the intracellular sequestration of GSH-conjugated toxins or metabolites in mammals^68^. Based on the evidence presented above, ATPase7A, ZnT1 and MRP appear to be the most likely candidates for Cd transport in oysters.

### Gene family expansion and positive selection

**Figure 6:**
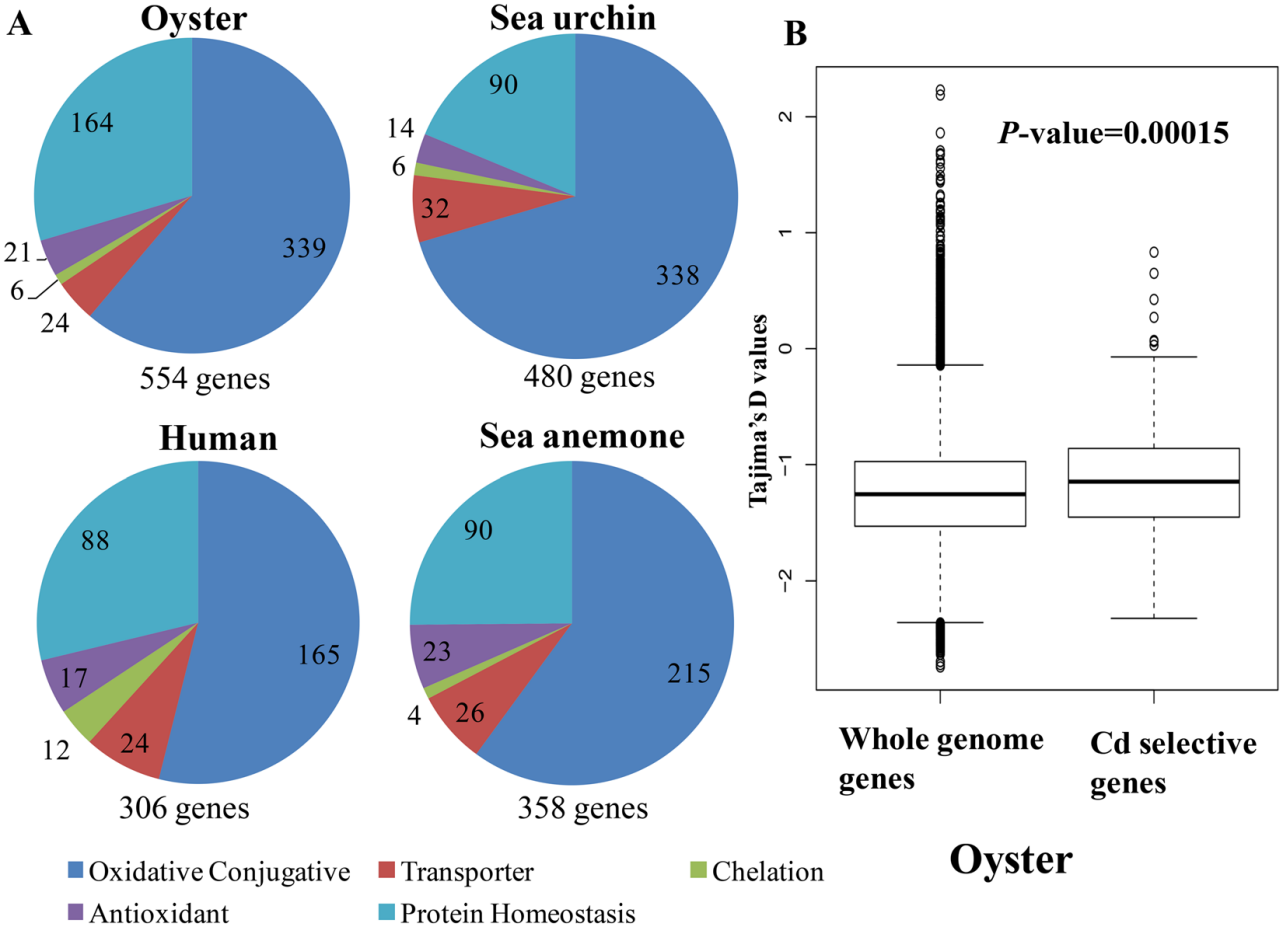
Expansion and positive selection analysis of Cd response genes in oysters. (**A**) Number of Cd responsive genes in the human, sea anemone, sea urchin, and oyster genomes. (**B**) Comparison of Tajima’s D values between Cd-selective genes (including the gene families of HSP70, EcSOD and CYP450) and the whole genome in oyster. Significance was assessed through the Wilcoxon test with *P* < 0.01 indicating significant differences.

The gene family expansion has been conducted in our previous studies^14^. Based on a previous study on *C. gigas*, we searched for evidence of gene family expansion of metal responsive genes by dividing responsive genes into five classes: metal chelation, transportation, protein homeostasis, antioxidant enzymes and oxidative conjugative processes. These related gene families were compared among four different species: C. gigas, *Homo sapiens*, *Nematostella vectensis*, and *Strongylocentrotus purpuratus*. Results suggested that oyster had 554 genes for metal-responsive processes, whereas only 306, 480 and 358 genes were found in human, sea urchin and anemone genomes, respectively (Fig. 6A). Further, HSP70, SOD and cytochromes (CYP) 450 showed expanded gene numbers involving protein homeostasis, antioxidant processes and oxidative comjugative processes (Table S6).

Excess Cd can indirectly produce reactive radicals, which lead to oxidative stress and altered protein metabolism. Our results also revealed that SOD and HSP participate in Cd defense system. The expanded number of SOD and HSP70 genes and the high expression levels found by us indicate that oysters have evolved strong defenses against oxidative stress and for maintaining protein homeostasis in the face of metal exposure. CYP450 is a key process in many detoxification pathways and its role in metal detoxification has been demonstrated in previous studies^69^, but not in molluscan. In our study, one CYP450 displayed increased protein abundances under Cd exposure (Table S5), indicating an important roles in Cd defense. Combined with its expanded gene numbers, we proposed that oysters have powerful CYP450 system, which may also play an important role in the Cd defense system.

The Tajima’s D values that were calculated for the expanded gene families and the whole genome gene set using the resequencing dataset for oysters *C. gigas* ^70^. The results indicate that candidate genes Tajima’s D values were significantly higher than the whole genome genes in oysters (Fig. 6B), revealing the positive selection on the expanded genes. These evolutionary changes in the gene numbers of these key gene families could reflect the oyster’s environmental adaptation.

## Conclusion

Oysters accumulate high Cd concentrations in their body tissues, and our study provides a detailed insight into the molecular mechanisms at the transcription and protein expression level involved in Cd effects on oysters. Excess Cd induced excess ROS, which may have caused oxidative stress and affected DNA and protein metabolism. Regarding DNA metabolism, Cd decreased the DNA repair capacity, reflected by the inhibition of the mRNA and protein expression of DNA glycosylase and gap-filling and ligation enzymes in the short-patch BER pathway. Regarding protein metabolism, Cd induced HSP responses, the initiation of protein refolding, and degradation by the ubiquitin proteasome pathway, among other effects. Cd also induced antioxidant enzyme responses and affected GSH metabolism, which is one of the most important antioxidant agents. A correlation analysis of the DEGs and DAPs, as identified in the GO annotation, gave close agreement between upregulated or downregulated genes (at mRNA level) and protein abundance, with a correlation coefficient of 0.72 (Fig. S8), indicating relatively strong consistency in responses. However, it should be kept in mind that the present study focused on only a sub-set of numerically important and significantly changed gene/protein responses under Cd exposure, whereas other genes/proteins were not studied.

## Materials and Methods

### Oyster culture

Oysters (*Crassostrea gigas*) were purchased from Yan Dunjiao aquaculture farm in Weihai, Shandong Province, China, and placed in seawater tanks for one week before the experiments. During acclimation and Cd exposure, the oysters were maintained in filtered seawater (pH 8.1 ± 0.1, salinity 30 ± 1) at 18 ± 2 °C and fed with a restricted quantity (0.05 g) of freshly cultured microalga (*Isochrysis galbana*) every other day, in order to minimize metal transfer via a trophic route.

The oysters were transferred to six 20-L filtered-seawater tanks (20 oysters per tank). CdCl_2_ was dissolved in deionized water to obtain a Cd stock solution (1 mg/L), which was then added to the six tanks to obtain a final nominal Cd concentration of 6.0 µg/L. For sample collection, six oyster gills were collected for each time point (0 h and 9 h), by sacrificing 2 individuals taken from 3 tanks for each time point. They were immediately frozen in liquid nitrogen before being stored at −80 °C. Gills from three individuals (100 mg) from three tanks were homogenized and used for protein extraction, which form one biological replicate. Two replicates were used for iTRAQ analysis. The seawater exposure group was used as the control. In each tank, the seawater (100%) was changed every two days, and the pH and salinity were checked and adjusted.

### Cd concentration and reactive oxygen species (ROS) detection

Gill homogenates of similar weights were freeze-dried and thoroughly digested in concentrated HNO_3_ (5 mL) and HCl (1 mL). This mixture was boiled, and after cooling, Cd levels in the gills were determined by inductively coupled plasma-mass spectrometry using an Agilent 7700x (Agilent Technologies, CA, USA)^71^. We measured ROS production in the gills under Cd exposure (0 h and 9 d) according to the 2′,7′-dichloro fluoresceindiacetate (DCFH-DA, Sigma) method using the commercial kit (Nanjing Jiancheng Bioengineering Institute, Nanjing, China).

### Activities of antioxidant enzymes activities and GSH, GSSG content

Gills (0.1 g) of oysters after Cd exposure (0 h and 9 d) were collected and rinsed twice with cold phosphate buffered saline (PBS), then lysed by glass tissue homogenizer in 900 µl PBS on ice. The homogenate was centrifuged at 3,500 × g for 10 min at 4 °C to precipitate insoluble material. The supernatant was collected and assayed for GR, GST, GPX activities and GSH, GSSG contents using kits (Nanjing Jiancheng Bioengineering Institute, Nanjing, China), following the manufacturers specifications^72^. The GPX, GST and GR activities were expressed as units/g protein, GSH content was expressed as µmol/g protein. One unit of GR activity was defined as the amount of enzyme depleting 1 mmol NADPH in 1 min; One unit of GST activity was defined as the amount of enzyme depleting 1 μmol GSH in 1 min; One unit of GPX activity was defined as the amount of enzyme depleting 1 μmol GSH in 1 min. At each time point (0 h and 9 d), six replicates were used for measurement.

### Protein preparation

The protein preparation procedure was conducted as in previous reports and was slightly modified^73^. The protein content was quantified with Bradford Protein assay kit (Beyotime Institute of Biotechnology, Jiangsu, China). In each group (control and Cd exposure group), six oysters were prepared and form two replicates. For each replicate, six oysters were processed to give two replicates and ground in powder in liquid nitrogen. Then, the powder was added to lysis buffer (7 M urea, 2 M thiourea, 4% CHAPS and 40 mM Tris-HCl, pH 8.5) and 10 mM dithiothreitol (DTT) (final concentration)^74^. The suspension was sonicated and centrifuged, and the supernatant was added to a 5-fold volume of chilled acetone containing 10% (v/v) trichloroacetic acid overnight at −20 °C. After centrifugation, the supernatant was discarded. The precipitate was then dissolved in lysis buffer (7 M urea, 2 M thiourea, 4% NP-40 and 20 mM Tris-HCl, pH 8.0–8.5), 10 mM DTT and 55 mM IAM. In order to block cysteine, the solution was incubated in the dark for 1 h. The chilled acetone was then added to the supernatant for 2 h to precipitate the proteins. The pellet was diluted in 500 μL 0.5 M tetraethylammonium bromide (TEAB) buffer (Applied Biosystems, Milan, Italy) and sonicated for 15 min. Finally, the samples were centrifuged and the proteins stored at −80 °C until analysis.

### iTRAQ labelling and strong cation exchange (SCX) fractionation

The proteins (100 μg) were digested with 3 μL trypsin gold for 16 h (protein:trypsin, 30:1). The peptides were reconstituted in 0.5 M TEAB and processed according to the manufacturer’s protocol for 8-plex iTRAQ reagent (Applied Biosystems). The labelled mixtures were then reconstituted with buffer A (25 mM NaH_2_PO_4_ in 25% acetonitrile (ACN), pH 2.7) and loaded onto an Ultremex SCX column containing 5 μm particles (Phenomenex, CA, USA). The peptides were eluted with a gradient of buffer A for 10 min, 5–60% buffer B (25 mM NaH_2_PO4 and 1 M KCl in 25% ACN, pH 2.7) for 27 min, and 60–100% buffer B for 1 min. Elution was monitored by measuring the absorbance at 214 nm, and fractions were collected every 1 min^75^.

### Liquid chromatography-electrospray ionization-tandem mass spectrometry analyses

Each fraction was resuspended in buffer A (5% ACN and 0.1% FA) and centrifuged for 10 min; the average final peptide concentration was 0.5 μg/μL. The supernatant was loaded onto a 2 cm C18 trap column on a LC-20AD nano-HPLC system (Shimadzu, Kyoto, Japan). The peptides were then eluted on a 10 cm analytical C18 column (inner diameter, 75 μm). The samples were loaded as follows: 8 μL/min for 4 min; 35-min gradient running at 300 nL/min; a 5-min linear gradient to 60%; a 2 min linear gradient to 80%; 80% B for 4 min and 5% for 1 min. Data acquisition was performed using a TripleTOF^®^ 5600 system (Sciex, Concord, ON, Canada) that was fitted with a NanoSpray^®^ III source and a pulled quartz tip as an emitter^75^.

### Protein identification

Raw data files acquired from an Orbitrap TripleTOF^®^ 5600 system were converted into MGF files (5600 ms converter) using Proteome Discoverer^™^ 1.2 (Thermo Fisher), which were then searched. Protein identification was performed using Mascot (http://www.matrixscience.com/) against a database containing 26,086 protein sequences (http://www.oysterdb.com). The parameters was set as: a mass tolerance of 0.05 Da (ppm) was permitted for intact peptide masses and 0.1 Da for fragmented ions, with allowance for one missed cleavage in the trypsin digests. The charge states of the peptides were set to + 2 and + 3. An automatic decoy database search was performed in Mascot by checking the decoy checkbox, in which a random sequence of databases is generated and tested for raw spectra, as was the real database. To reduce the probability of false peptide identification, only peptides with significant matching scores value (matching between observed mass value and theoretical mass ≥20 at the 99% confidence interval by a Mascot probability analysis) were counted as identified. The Mascot score was calculated as −10log(10) (*P*-value), with *P* being the absolute probability^76^. The Mascot score of 20 means that the peptide spectrum match probability was 1% (*P*-value = 0.01), which was also used in previous iTRAQ analysis^73,77^.

The proteins containing at least two unique peptides were used for quantitation. Protein quantitation was performed at the peptide level using Mascot (http://www.matrixscience.com/). The peptide matches to protein hits, and the median peptide ratio were selected to represent the protein ratio. When an even number of peptide ratios was encountered, the geometric mean of the median pair was used. The protein ratio *p*-values were calculated according to the procedures described in http://www.matrixscience.com/ help/quant_statistics_help.html and *t*-test was performed using the Mascot software. In our results, the selection criterion for DAPs was: the quantitative signals with *P*-vale ≤ 0.05 and protein ratio (Cd exposure group/control group) ≥1.2 (≤0.83), which have been widely used in many previous studies^78,79^.

### Functional annotation analysis

A GO functional annotation of the proteins was conducted using the Blast2GO program in the non-redundant protein database (NCBI)^80^. The Kyoto Encyclopaedia of Genes and Genomes (http://www.genome.jp/kegg/) and COG (http://www.ncbi.nlm.nih.gov/COG/) databases were also used for protein identification and classification^31^.

### Protein extraction, preparation of antibody, and western blotting analysis

Gills of oysters from the different time points (Cd exposure 0 h and 9 d) were thawed in ice and homogenized with ice-cold phosphate-buffered saline (PBS), then centrifuged at 5000 *g* and 4 °C for 30 min. The supernatants were collected, quantified according to the Bradford Protein assay kit (Beyotime Institute of Biotechnology, Jiangsu, China), and adjusted to the same concentration for the following assays.

For the preparation of antibodies against CgMT2, renatured CgMT2 protein was dialyzed continuously against ddH_2_O before being freeze concentrated. Western blotting was performed according to published methodology^81^. The CgMT2 proteins were presented to 6-week-old rats to acquire polyclonal antibody. Protein extracts of the samples (Cd exposure 0 h and 9 d) were added in a 5× protein loading buffer and denatured by boiling for 10 min. After sodium dodecyl sulfate (SDS)-polyacrylamide gel electrophoresis (PAGE), the samples were electrophoretically transferred onto a 0.45-mm pore nitrocellulose membrane at 250 mA for 3 h. The membranes were blocked with PBS containing 5% skim milk powder at 37 °C for 1 h. After three washes with PBS containing 0.05% Tween-20 (PBST), the membranes were incubated with the primary antibodies (1:1000 dilutions in blocking solution) at 4 °C for 12 h. The primary antibodies consisted of anti-CgMT2 antibodies, as well as anti-α tubulin antibodies (Abcam, ab15246). After three washes with PBST, the membranes were incubated with goat anti-rat IgG-HRP or goat anti-rabbit IgG-HRP (1:3000–1:5000 dilutions in PBS) for 1.5 h at room temperature with shaking and then washed with PBST three times. The membranes were exposed to X-ray film and the visualized. The band intensity was quantified using Quantity One software (Bio-Rad, USA).

### Transcriptome analysis under Cd exposure

The transcriptome data were submitted to the NCBI database with the GEO number GSE31012. Two-year-old Pacific oysters (shell length, 9–12 cm) were purchased from a farm in Weihai, China in January 2011. After one week of acclimation, three replicate tanks containing 120 individual oysters were exposed to dissolved Cd (6 µg/L). After 0 h and 9 days of Cd exposure, three oyster gills in each tank were collected and a total of nine individuals from three tanks were mixed and used for RNA extraction at each time point. RNA was extracted individually using Trizol reagent (Invitrogen, USA). RNA collected from each individual at the same time point were mixed equally for RNA-seq analysis using an Illumina HiSeq. 2000 platform. Salinity (30 ± 1), temperature (20 ± 0.5 °C) and pH (8.0 ± 0.3) were maintained throughout the experiments.

### Gene expression value measurement of transcriptome

Gene expression profiling was measured by mapping reads to assembled sequences using Tophat^82^. Gene expression level was measured by RPKM (Reads Per Kilobase per Million mapped reads)^83^. To exclude the bias caused by different RNA output between samples, the TMM (trimmed mean of M values) method to calculate a normalization factor introduced by Robinson was adopted^84^, using the calcNormFactors function in edgeR package^85^.

### Differentially expressed genes of transcriptome

We modified Audic’s method to analyze differential expression^86^. Audic’s method was used originally for pairwise comparisons of cDNA libraries. The formula to calculate the significant *P*-value is defined as Chen *et al*.^87^:

1 $${\rm{C}}({\rm{y}}\le {\text{y}}_{\text{min}}|{\rm{X}})=\sum _{y=0}^{y\le y\,\min }p(y|x)$$

2 $${\rm{D}}({\rm{y}}\ge \,{{\rm{y}}}_{\min }\,|{\rm{X}})=\sum _{y=y\,\max }^{\infty }p(y|x)$$

3 $$p(y|x)={(\frac{N2}{N1})}^{y}\frac{(x+y)!}{x!y!{(1+\frac{N2}{N1})}^{(x+y+1)}}$$

In formula (1), (2) and (3), x and y were defined as the mapped reads per kilobase of a transcript to avoid bias of sequence length, whereas N1 and N2 represent the total mapped read counts of two compared sequence database. The FDR (false discovery rate) cutoffs method was used to control the *P*-value. Finally, the differentially expressed genes were set as: FDR < 0.01. The fold changes were calculated as: (RPKM_Cd 9d exposure_)/(RPKM_control_).

### Gene family expansion and positive selection

Gene family expansion analysis was conducted as described in a previous study by us^14^. To assess the expansion of gene families related to metal defense, oyster genes were compared to those of the cnidarian *Nematostella vectensis* and to those of the echinoderm *Strongylocentrotus purpuratus*, which have a similar marine habitat as *C. gigas*, as well as *Homo sapiens*, which has the best studied reference genome. Oyster genes were identified based on genome sequence domain structures. Proteins from reference species were searched against the oyster protein database using the protein-protein basic local alignment search tool (BLASTP). Then these genes were blasted against NCBI-nr and Swiss-Prot databases for annotation. In all these species, proteins shorter than 50 amino acids were excluded. According to these analysis, we obtained the gene numbers in each family. Other gene number data from a previous study was included^14^. We tested whether *C. gigas* had significantly more genes in each selected family than other species using chi-square test. The number of all annotated genes of each species were used as the backgrounds. *P*-values from both chi-square tests are presented.

Ten individuals of *C. gigas*, used in a previous study^70^, were re-sequenced (raw data submitted to GenBank as BioProject SRP056090) for determining positive selection. Paired-end resequenced reads were mapped to the reference genome using Bowtie. Single nucleotide polymorphisms (SNPs) calling was then performed on a population scale using the Bayesian approach implemented in the package SAMtools^59^. A sliding window approach was applied to quantify polymorphism level. Values of Tajima’s D (a measure of selection in the genome) and polymorphism level were compared between whole genome genes and the metal-response candidate gene sets. The same positive selection method has been widely used in many previous studies^70,88^. The statistical differences were assessed by a Wilcoxon test (*P* < 0.01).

### Ethic statement

All the methods were carried out in accordance with the approved guidelines. In addition, all the experimental protocols were approved by a safety committee.

### Data Accessibility

Whole oyster genome sequences were obtained from http://gigadb.org/dataset/100030. The proteomics data were submitted to Proteome X change (PXD003290).

## Electronic supplementary material

supporting information

Table S4

Table S3
