# Supplementary material for: Cadmium effects on DNA and protein metabolism in oyster (Crassostrea gigas) revealed by proteomic analyses
Source: Sci Rep. 2017 Sep 15;7:11716. doi: 10.1038/s41598-017-11894-7 (PMC5601910; doi:10.1038/s41598-017-11894-7)
Supplement: Supplementary file 1 — supporting information [file 41598_2017_11894_MOESM1_ESM.pdf]

Cadmium effects on DNA and protein metabolism in oyster (*Crassostrea gigas*) revealed by proteomic analyses

Jie Meng[1,3,4], Wen-Xiong Wang[5*], Li Li[1,3,4*], Qi Yin[6] & Guofan Zhang[1,2,4]

[1] Key Laboratory of Experimental Marine Biology, Institute of Oceanology, Chinese Academy of Sciences, Qingdao 266071, Shandong, China
[2] Laboratory for Marine Biology and Biotechnology, Qingdao National Laboratory for Marine Science and Technology, Qingdao 266071, Shandong, China
[3] Laboratory for Marine Fisheries and Aquaculture, Qingdao National Laboratory for Marine Science and Technology, Qingdao, Shandong, China
[4] National& Local Joint Engineering Laboratory of Ecological Mariculture, Qingdao 266071, Shandong, China
[5] Marine Environmental Laboratory, HKUST Shenzhen Research Institute, Shenzhen 518057, China
[6] BGI-Shenzhen, Shenzhen 518083, China

[*]Corresponding authors: Wen-Xiong Wang (wwang@ust.hk)

Li Li (lili@qdio.ac.cn)

This file includes:
Supplementary figures S1-S8.
Supplementary tables S1-S7.

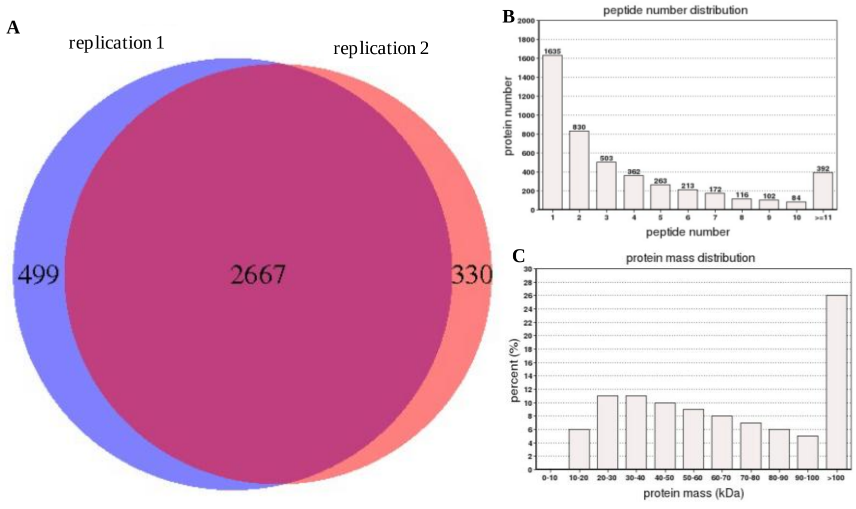

Fig. S1. The quantified proteins numbers (A), peptide number distribution (B) and protein mass distribution of iTRAQ database.

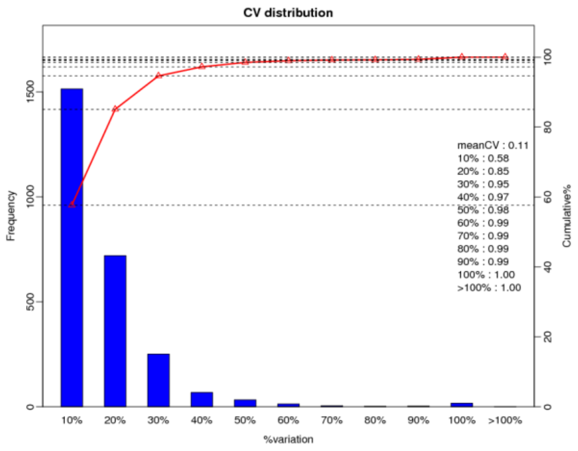

Fig. S2. The coefficients of variation (CV) analysis between two replications under 9 d of Cd exposure.

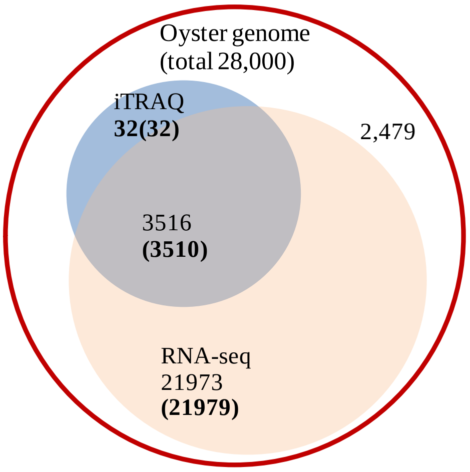

Fig. S3. The gene/protein numbers in RNA-seq, iTRAQ and oyster genome database. The iTRAQ data obtained from two replicates were used for this analysis. The data in the parentheses represent the second replicate.

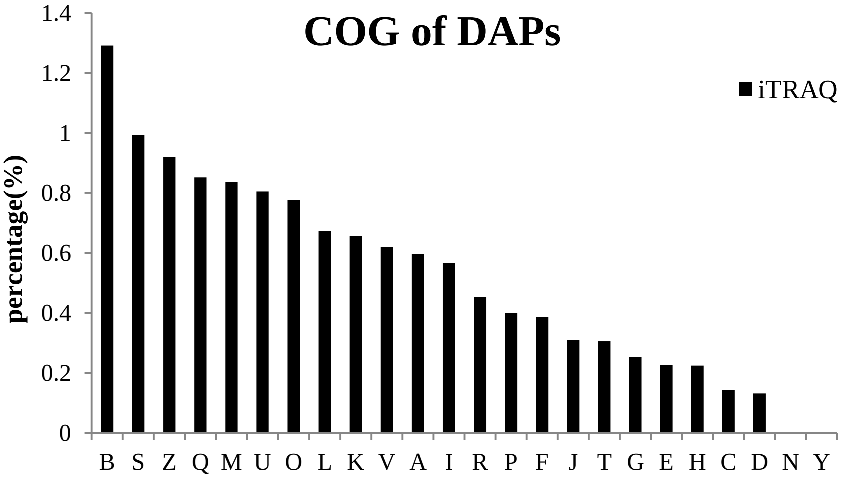

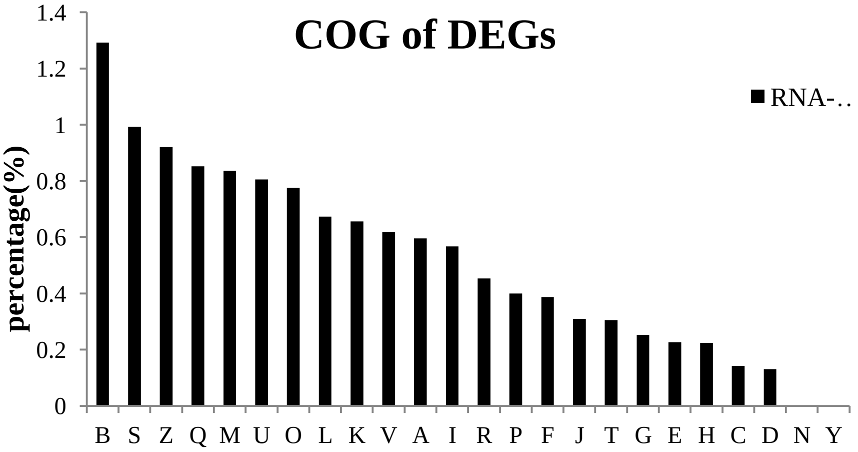

Fig. S4. The COG analysis of the DAPs and DEGs identified in iTRAQ and RNA-seq database. A: RNA processing and modification; B: Chromatin structure and dynamics; C: Energy production and conversion; D: Cell cycle control, cell division, chromosome partitioning; E: Amino acid transport and metabolism; F: Nucleotide transport and metabolism; G: Carbohydrate transport and metabolism; H: Coenzyme transport and metabolism; I: Lipid transport and metabolism; J: Translation, ribosomal structure and biogenesis ; K: Transcription; L: Replication, recombination and repair; M: Cell wall/membrane/envelope biogenesis; O: Posttranslational modification, protein turnover, chaperones; P: Inorganic ion transport and metabolism; Q: Secondary metabolites biosynthesis, transport and catabolism; R: General function prediction only; S: Function unknown; T: Signal transduction mechanisms; U: Intracellular trafficking, secretion, and vesicular transport; V: Defense mechanisms; Z: Cytoskeleton

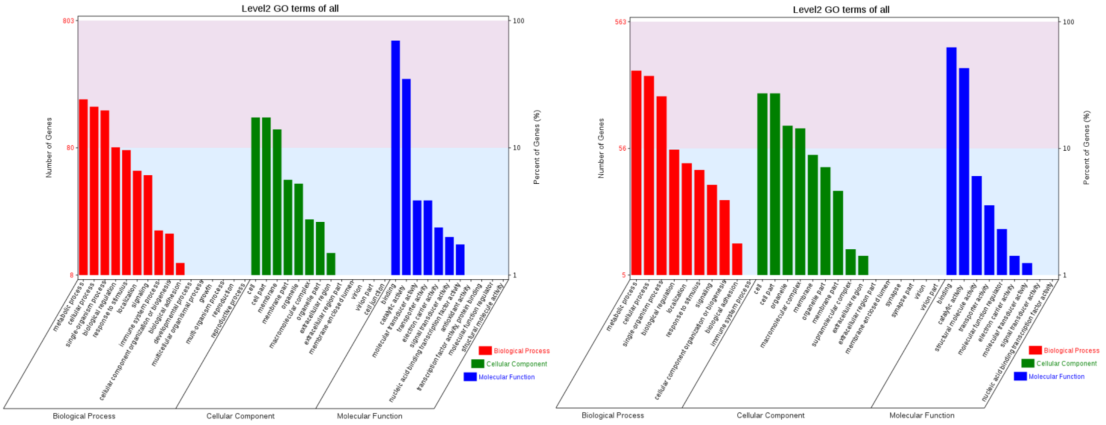

Fig. S5. The GO analysis (level 2) for both RNA-seq (A) and iTRAQ data (B).

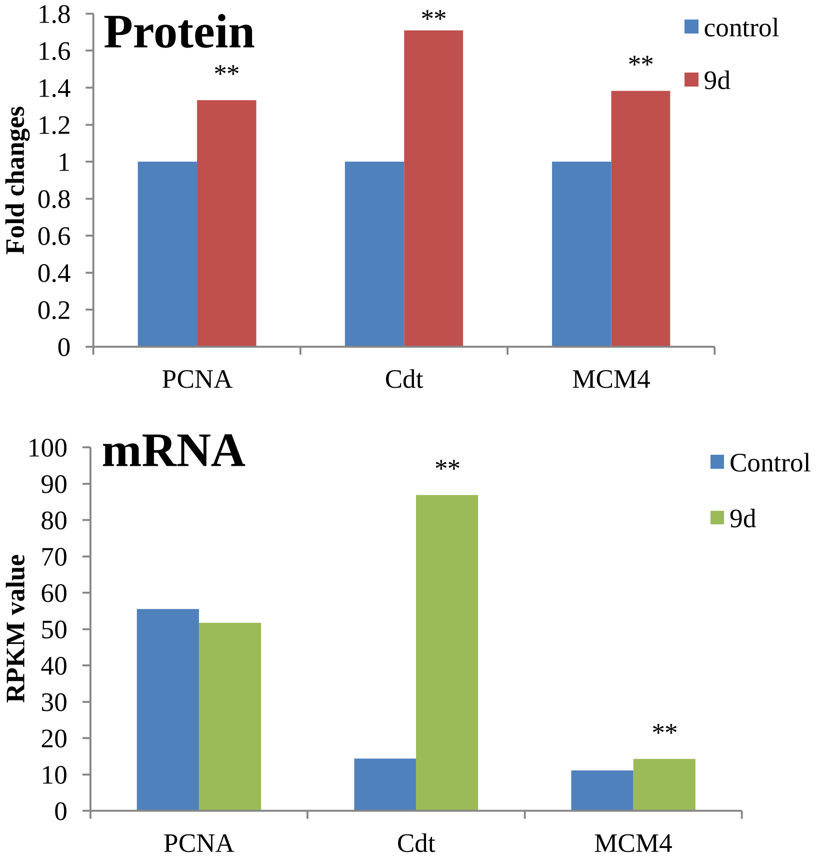

Fig. S6. The mRNA expressions (A) and protein fold changes (B) of DNA damage related genes/proteins after 9 d of Cd exposure. The mRNA expressions were measured by RPKM (reads per kilobase per million mapped reads) from RNA-seq data. The significance was calculated with Chen's methods, with FDR$<$0.01 (**). For protein abundance, the fold changes compared with control group (0 h after exposure) after 9 days of Cd treatment were displayed (*indicates $P \leq 0.05$ and fold changes $\geq$1.2, n=2).

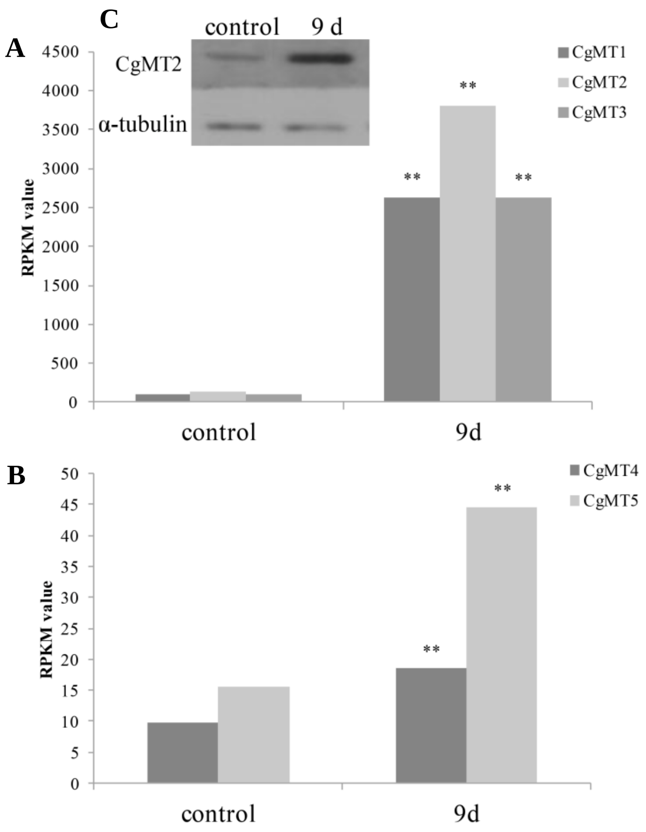

Fig. S7. The mRNA expression and protein abundance of metallothionein in control and Cd exposure group. (A, B) The mRNA expressions were measured by RPKM (reads per kilobase per million mapped reads) from RNA-seq data. The significance was calculated with Chen's methods, with FDR$<$0.01 (**). (C) Western blot analysis of CgMT2 in oyster under control and 9 d of Cd exposure. The α-tubulin were used as the reference protein.

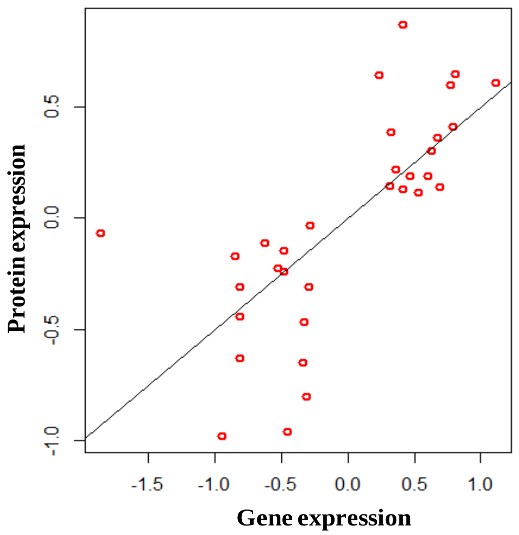

Fig. S8. The Correlation analysis between transcriptional and translational data.

Table S1: The characteristic of iTRAQ database.

Group name	Total spectra	Spectra	Unique spectra	Peptide	Unique peptide	Protein
**replication 1**	421868	55135	50089	20919	19863	4672
**replication 2**	365464	56996	51700	21425	20270	4964

Table S2: The characteristic of RNA-seq database

Sample ID	*RPKM > 0	RPKM ≥ 1	RPKM ≥ 5	#Total reads (M)	%Mappable
Control	24201	20713	15269	17.17	69.10%
Cd_9d	23814	20504	15215	16.84	70.92%

*RPKM represents the gene expression value. RPKM value = (Reads Per Kilobase per Million mapped reads)

Table S3 GO analysis of Cd responsive proteins after 9d treatment using iTRAQ data (biological process, $P < 0.05$).

See excel file

Table S4 GO analysis of Cd responsive genes after 9d treatment using RNA-seq data (biological process, $P < 0.05$).

See excel file

Table S5 Antioxidant enzyme downregulation at mRNA (DEG) and protein abundance (DAP), after 9d Cd exposure. Red shading indicates significantly changed genes/proteins (DEGs or DAPs). Units for protein response correspond to -fold changes relative to control, units of mRNA correspond to RPKM value of control and Cd exposure group, respectively. The significance was calculated with Chen's methods, with FDR＜0.01. For iTRAQ relative quantification analysis, the value represent the ratios of protein abundance in the 9 d Cd exposure treatment compared to the control treatment ($P \leq 0.05$ and fold changes ≥1.2, n=2).

Gene names	Protein		mRNA	
	GeneID	9d	Control	9d

CAT	CGI_10003354	0.895	34.46	58.24
Cu/Zn-SOD	CGI_10004092	0.804	1684.51	968.15
Cu/Zn-SOD	CGI_10018834	0.768	152.55	112.16
Cu/Zn-SOD	CGI_10026148	0.841	2243.68	1273.38
Mn-SOD	CGI_10017307	0.791	89.52	39.92
CYPP450 18a1	CGI_10002510	1.730	59.78	79.64

Table S6 Gene families numbers encoding Cd responsive related genes in oyster genome with other selected sequenced genome.

	**Gene**	**Sea anemone**	**Oyster**	**Sea urchin**	**Human**
[14]Antioxidant	Superoxide dismutase (SOD)	4	**6****	6	3
	Catalase (CAT)	1	2	1	1
	Peroxidase (PRX)	6	5	3	5
	Glutathione peroxidase (GPX)	12	8	4	8
[14]Protein Homeostasis	heat shock protein 70(HSP70)	12	**88****	40	14
	Heat shock protein 90(HSP90)	2	2	3	9
	Heat shock protein 20(HSP20)	14	18	4	10
	Heat shock protein 60(HSP60)	21	16	17	16
	Heat shock protein DnaJ	41	40	26	39
Transporter	Zinc transporter	26	24	32	24
Chelation	Metallothionein(MT)	0	5	3	12
	Phytochelatin synthase(PCs)	4	1	3	0
Oxidative Conjugative	CYP450	82	**136****	120	57
	FMO	6	14	16	6
	ALDH	21	22	20	19
	EPHX	1	1	5	2
	GST	18	35	38	18
	SULT	22	63	73	13
	NAT	0	0	1	2
	ABC transporter	65	68	65	48
Total		215	339	338	165

The ** means the *P*-value ＜0.01 which was obtained from Chi-square test for overrepresentation in *C. gigas* genome, and all annotated genes were used as the

background. Gene families with superscripted number were identified in previous study [14].

Table S7 Genome data used in gene family analysis

**Species Name**	**Source**	**File Name / Version**	**predicted genes**
Nematostella vectensis	JGI	http://genome.jgi-psf.org/Nemve1	37,088
Homo sapiens	ENSEMBL	http://ensemblgenomes.org/info/genomes	22,389
Strongylocentrotus purpuratus	SpBase	http://spbase.org/	29,122
Crassostrea gigas	OysterdataBase	http://www.oysterdb.com	28,027
